# Genetic Variations in VDR could Modulate the Efficacy of Vitamin D_3_ Supplementation on Inflammatory Markers and Total Antioxidant Capacity among Breast Cancer Women: A Randomized Double Blind Controlled Trial

**DOI:** 10.31557/APJCP.2019.20.7.2065

**Published:** 2019

**Authors:** Houra Mohseni, Reza Amani, Seyed Ahmad Hosseini, Alireza Ekrami, Ahmad Ahmadzadeh, Seyed Mahmoud Latifi

**Affiliations:** 1 *Department of Nutrition, Faculty of Paramedicine, *; 2 *Diabetes Research Center, *; 4 *Nutrition and Metabolic Disease Research Center, *; 5 *Infectious and Tropical Diseases Research Center,*; 6 *Thalassemia and Hemoglobinopathy Research Center, Research Institute of Health, Ahvaz Jundishapur University of Medical Sciences, Ahvaz, *; 3 *Food Security Research Center, Department of Clinical Nutrition, Isfahan University of Medical Sciences, Isfahan, Iran. *

**Keywords:** Breast cancer- inflammation- supplementation- VDR polymorphisms- Vitamin D_3_

## Abstract

**Background:**

Low levels of vitamin D are found in a great part of breast cancer women. Study subjects using vitamin D_3_ supplement had lower rates of cancers and fewer markers of inflammation. Additionally, recent studies demonstrate the power of vitamin D supplementation to lower inflammation and oxidative stress biomarkers associate with VDR polymorphism to reduce inflammation. This study was aimed to assess the impact of vitamin D_3_ supplementation on the serum concentration of inflammatory markers and antioxidant capacity with regard to VDR polymorphism in the VDR gene in breast cancer women.

**Methods:**

A randomized, double-blind, placebo-controlled trial was conducted on 56 breast cancer women. Participants were assigned to 2 treatment arms: placebo and vitamin D_3_ for 2 months intervention. Supplementation group received 50,000 IU of vitamin weekly. Blood samples were collected at baseline and after the intervention to measure the 25(OH) D_3_, TNF-α, TGF- β and TAC. Genotyping was performed for FokI, BsmI, ApaI, and TaqI polymorphism.

**Results:**

After eight weeks supplementation, the intervention group showed a significant increase in the serum concentration of 25(OH) D_3_ (28±2.6 to 39±3.5; p=0.004 and TAC (48.9±13.3 to 63.5±13.3; p= 0.017). Changes in TNF-α, TGF- β1 were not significant. Serum TAC levels of participants with the TT/Tt, Ff genotypes were more responsive to supplementation**. **

**Conclusions:**

Supplementation with a vitamin D_3_ increased the TAC in breast cancer women, although it had no effect on inflammatory markers. Serum TAC in the TT/Tt, Ff were more responsive to vitamin D supplement compared with those with the FF/ff and tt genotypes.

## Introduction

The vitamin D system comprises a group of fat-soluble prohormones and their various metabolites. It has been eminent for its role in calcium homeostasis and maintenance of bone metabolism (Holick, 2007). Low levels of vitamin D are found in a great part of the population leading some authorities to state a worldwide epidemic of vitamin D deficiency and to recommend vitamin D supplementation. Concerns about vitamin D deficiency rose when further research revealed cancer patients had lower levels of serum 25(OH)D (25-hydroxyvitamin D), and study subjects using vitamin D had lower rates of cancers and fewer markers of inflammation (Neuhouser et al., 2008; Mohr et al., 2014). Considerable data propose that the adding calcitriol to several chemotherapy regimens intensify the activity of such treatments and possibly a better response level to the regimens (Ma et al., 2010).

Studies suggested that inflammation mediates the initiation and progression of tumors, angiogenesis, and metastasis in patients with cancer. Epidemiological studies have shown that low 25(OH)D levels are associated with higher levels of systemic inflammation (Ngo et al., 2010; Crowley, 2014). Additionally, recent studies demonstrate the power of vitamin D supplementation to lower inflammation and oxidative stress biomarkers, by operating at the DNA level to regulate genes to reduce inflammation (Chen et al., 2014). These data remain debated, a recent meta-analysis had no success to show the helpful effects of vitamin D supplementation on inflammatory cytokines (Jamka et al., 2015). Moreover, in an animal study, vitamin D deficiency increased over the three-fold burden of liver tumor growth in the context of TGF-β (Transforming growth factor beta 1) (Chen et al., 2016). In another survey, TAC (total antioxidant capacity) levels decrease 35% in the serum of patients with cancer breast undergoing chemotherapy (Omar et al., 2011). 

Vitamin D performs its biological activities through binding to the VDR (vitamin D receptor), a particular high-affinity receptor. VDR is a member of nuclear receptors for steroid hormones and controls gene expression as a ligand-activated transcription factor (2012). The VDR gene has more than 460 SNPs (single nucleotide polymorphisms) that have the regulatory role on 1, 25(OH)_2_D_3_. Hence, they can be assumed as a predictor of cell reply to supplementation in different conditions (Abd-Elsalam et al., 2015). Four types of VDR polymorphisms consisting FokI, BsmI, ApaI, and TaqI have more association with breast cancer (Hutchinson et al., 2000; Newcomb et al., 2002). Therefore, it is predicted that increasing 25(OH)D may improve the concentrations of TAC parallel with reducing blood inflammatory markers.

A clinical trial showed that 4 weeks supplementation with 200,000 IU of vitamin D_3_ administered as a single dose improved the serum 25(OH)D and TAC levels and significantly lower the us-CRP (Ultra-Sensitive CRP) levels in old females with vitamin D insufficiency. The serum concentrations of 25(OH)D and us-CRP of participants with the BB/Bb genotype were more susceptible to supplementation (de Medeiros Cavalcante et al., 2015). An epidemiological study examined the risk of breast cancer related to polymorphism of VDR gens and 25 (OH)D in plasma. TaqI, one of the VDR polymorphism, reduced the risk of breast cancer by 26% (Reimers et al., 2015). In another study serum levels of 25 (OH)D of individuals with genetic variation of vitamin D receptor protein responded differently to vitamin D_3_ supplements (Nimitphong et al., 2013).

Despite the epidemiological evidence, there are no published trials regarding the effects of vitamin D supplementation on blood levels of 25 (OH)D, TNF-α, TGF-β1, and TAC in accordance with the different variation of VDR polymorphism among breast cancer women. Based on this purpose, we conducted a randomized, double-blind, placebo-controlled trial of vitamin D_3_ supplementation to estimate the changes of total 25 (OH)D over 2 months based on VDR genotypes.

## Materials and Methods

*Study population*

Who were followed up at the oncology ward, University Golestan Medical Center, between April to September 2015. Medical Research Ethics Committee at the Ahvaz Jundishapur University of Medical Science approved the study. Patients with metastatic breast cancer and those who had histories of other cancers, history of chemotherapy, radiotherapy, and hormone therapy for any reasons except current cancer, treating with corticosteroids, chronic diarrhea and malabsorption were excluded. Patients with known inflammatory conditions (such as acute bacterial or viral infections), autoimmune diseases (such as rheumatoid arthritis or lupus) were also excluded from the study. Patients were undergo chemotherapy at the beginning of study. The sample size of 23 was calculated for each group by considering significance level of P < 0.05 and power of 80% (Hopkins et al., 2011). Applying 20% dropout, a sample size of 28 for the placebo and intervention groups was yielded.

**Table 1:** Baseline Characteristics of the Clinical Trial Participants

	Treatment group		
Characteristics	Placebo	Vitamin D	p^a^
	n=26	n=26	
Demographics			
Age, y	46.3 (9.5)	47.7 (8.0)	0.6
Ethnicity,%			
Arab	54	43.5	
Fars	46	56.5	0.46
Breast cancer Stage, %			
I	33	27	
II	42	43	
III	25	30	0.5
Anthropometric			
BMI	29.2 ± 6.3	30.2 ± 5.4	0.59
Waist circumference	103.5 ± 12.5	109 ± 11.2	0.12
Mean dietary intakes			
Total energy intake, kcal/d	1,596 ± 528	1,848 ± 821	0.59
Total fat, g/d	67 ± 32	70 ± 32	0.59
Dietary calcium, mg/d	618 ± 308	843 ± 526	0.41
Dietary fiber, g/d	15 ± 7	18 ± 9	0.97
Dietary carotenoid intake (μg/day)	4743.74 ± 4771.63	4509.17 ± 3890.52	0.77
Dietary vitamin C intake (mg/day)	104.16 ± 79.14	108.80 ± 84.43	0.16
Dietary vitamin E intake (mg/day)	30.7 ± 18.28	29.8 ± 15.36	0.53
Dietary selenium intake (μg/day)	48.5 ± 29.77	46.9 ± 27.63	0.68

Data are given as means±SD unless otherwise specified;

a Chi square test was used for categorical variables; Independent t-test was used for continuous variables.

**Table 2:** Change in Biomarkers of Serum after Supplementation of Vitamin D in Breast Cancer Patients

Biomarkers	Placebo			Vitamin D			Absolute treatment effect a		
	Baseline	2 months	p c	Baseline	2 months	p^ c^	placebo	Vitamin D	P b
	follow up			follow up					
25 (OH) D (ng/mL)	15.3±2.2	13.4±2.2	0.59	28±2.6	39±3.5	0.004*	-1.9±0.9	11±3.1	0.001*
TNF-α (pg/mL)	32.6±8	25.6±3.2	0.19	13.4±1.1	14.5±1.6	0.96	7.0±2.1	1.1±2.1	0.18
TGF-β (pg/mL)	123.4±9	133.8±10	0.24	293.8±48.8	288±42.9	0.84	10.4±7.1	-5.6±33.4	0.64
TAC (U/ml)	45.2±11.5	29.2±8.3	0.001*	48.9±13.3	63.5±13.3	0.004*	-16±8.4	14.6±8.9	0.017*

Values are Mean±SD;

* p value < 0.05;

a Absolute treatment effect is the absolute change from baseline to follow-up in the treatment group minus the absolute change from baseline to follow-up in the placebo group;

b P values for differences between the treatment and placebo groups;

c P values for difference between baseline visit and post-intervention values; 25 (OH)D, 25-hydroxyvitamin D; TNF-α, tumor Necrosis Factor alpha; TGF- β1, transforming growth factor beta 1; TAC, total antioxidant capacity.

**Table 3:** Changes in TNF-α Levels with Regards to VDR Polymorphism in Breast Cancer Patient*

	Placebo				Vitamin D				Absolute treatment effect a		
Genotype	n	Baseline	2 months	p c	n	Baseline	2 months	p c	placebo	Vitamin D	P b
		follow up				follow up					
FokI											
FF	3	7.5±0.5	39.5±14.5	0.51	3	13.0±1.0	28.0±12	0.42	32.0±14.0	15.0±11	0.7
Ff	19	38.5±1.7	24.8±3.6	0.12	18	14±1.3	12.7±1.3	0.27	-13.7±9.9	-1.3±2	0.23
ff	4	11.67±1.2	20.67±8.4	0.27	5	8.5±1.5	18.0±5.0	0.24	9.0±9.0	9.5±3.5	0.34
BsmI											
BB	7	40.4±28.1	16.0±27	0.4	5	9.0±1.4	11.0±1.4	0. 4	-24.4±29.8	2.0±0	0.4
Bb	11	32.6±12	22.2±3.3	0.27	15	15.0±1.4	14.2±1.6	0.5	-10.4±11	-0.8±2.5	0.4
bb	8	27.7±8.2	36.0±7.4	0.5	6	10.5±1.4	19.2±7.0	0.3	8.3±9.0	8.7±6	0.97
ApaI											
AA	11	15.4±2.8	26.6±4.2	0.13	10	13.6±1.3	14.8±3.2	0.95	11.2±5.6	1.2±3.5	0.17
Aa	11	51.0±15.8	23.0±5.8	0.08	10	12.7±1.3	13.5±1.6	0.95	-28.0±15.4	0.8±2.0	0.094
aa	4	25.5±13.5	32.0±7.0	0.6	6	14.6±4.2	15.8±4.5	0.98	6.5±6.5	1.2±7.4	0.7
TaqI											
TT	8	21.0±6.2	15.8±3.9	0.4	7	16.5±4.6	11.5±1.2	0.32	-5.2±7.6	-5.0±5.1	0.98
Tt	15	41.6±12.2	29.4±4.7	0.3	13	13.7±1.0	15.9±16	0.68	-12.2±13	2.2±3	0.29
tt	3	14.0±2.6	22.6±5.3	0.5	6	10.8±2.0	13.4±3.0	0.69	8.6±7.6	2.6±4	0.53

* Values are Mean±SD;

* p value< 0.05;

a Absolute treatment effect is the absolute change from baseline to follow-up in the treatment group minus the absolute change from baseline to follow-up in the placebo group;

b P values for difference between treatment group and placebo group;

c P values for difference between follow-up visit and baseline visit; TNF-α, Tumor Necrosis Factor alpha; VDR, vitamin D receptor.

**Table 4:** Changes in TGF-β Levels with Regards to VDR Polymorphism in Breast Cancer Patient

	Placebo				Vitamin D				Absolute treatment effect a		
Genotype	n	Baseline	2 months	p c	n	Baseline	2 months	p c	placebo	Vitamin D	P b
		follow up				follow up					
FokI											
FF	3	169.6±45	183.6±39	0.31	3	245±125	277±127	0.18	14.0±14.0	32.0±2.0	0.55
Ff	19	116.8±8.6	124.3±10	0.5	18	293.8±58	289.5±51	0.89	7.5±8.5	-4.3±39.2	0.77
ff	4	116.5±8.5	150±30	0.38	5	341±60	285±65	0.72	33.5±21.5	-56.0±125	0.6
BsmI											
BB	7	116.4±5.8	140.6±21.4	0.3	5	220.5±305	280±80	0.69	24.2±18.7	59.5±110.5	0.8
Bb	11	146.8±12	148.3±14.6	0.9	15	313±67.7	320±59.4	0.88	1.5±4.2	7.1±43.2	0.9
bb	8	109.7±17	95.5±15.4	0.51	6	253±73.9	197±16.5	0.45	14.2±17.6	-56.0±67	0.37
ApaI											
AA	11	120.1±19.8	131.6±20.5	0.47	10	334±103	278±69	0.31	11.5±12.8	-56.0±53.0	0.25
Aa	11	123.8±3.4	134.0±10	0.41	10	225.5±30	239.2±34	0.7	10.2±9.6	13.7±32.6	0.91
aa	4	138.5±2.5	145.5±9.5	0.6	6	344±120	394±146	0.68	7.0±7.0	50.0±110	0.71
TaqI											
TT	8	140.8±30	139.4±31	0.6	7	431±229	347±154	0.4	-1.4±6.1	-84±87.0	0.41
Tt	15	111.7±9.4	124.5±11	0.33	13	284.2±52	282.6±56	0.94	12.8±10.8	-1.6±38.0	0.7
tt	3	146.7±22	164±31.9	0.5	6	222.9±29	260.5±66	0.68	17.3±19	37.6±84.2	0.87

* p value< 0.05;

a treatment effect is the absolute change from baseline to follow-up in the treatment group minus the absolute change from baseline to follow-up in the placebo group;

b P values for difference between treatment group and placebo group;

c P values for difference between follow-up visit and baseline visit; TGF- β1, transforming growth factor beta 1; VDR, vitamin D receptor.

**Table 5:** Changes in TAC Levels with Regards to VDR Polymorphism Following Vitamin D Supplementation in Breast Cancer Patient

Genotype	Placebo				Vitamin D				Absolute treatment effect a		
	n	Baseline	2 months	p c	n	Baseline	2 months	p c	placebo	Vitamin D	P b
		follow up				follow up					
FokI											
FF	3	51.6.±41	50.0±41.5	0.15	3	96.5.±86	164.5±5.5	0.59	-1.6±1.6	68.0±92	0.58
Ff	19	39.2±11.4	27.7±8.7	0.1	18	43.0±13.9	53.1±13.6	0.12	-11.3±7.8	10.1±5.7	0.036*
ff	4	92.0±81.0	10.0±4.0	0.48	5	54.5±38.5	56.0±37.0	0.79	-82±81.0	1.5±1.5	0.41
BsmI											
BB	7	48.8±25.6	46.6±22.5	0.42	5	114.0±91	120±99	0.64	-2.2±4.7	6.0±8.0	0.4
Bb	11	52.7±17.9	35.5±14.5	0.18	15	39.0±14.6	47.4±12	0.32	-17.2±13	8.3±7.1	0.08
bb	8	9.5±1.6	32.3±20.0	0.25	6	34.0±19.7	76±35.6	0.37	-22.8±20	42.0±39.3	0.12
ApaI											
AA	11	50.0±19.4	31.9±14.8	0.19	10	25.0±9.7	55.6±19.6	0.18	-18.1±14.5	30.6±19.9	0.06
Aa	11	46.1±16.2	29.1±11.0	0.19	10	56.8±26	64.4±25	0.38	17±13.5	7.6±7.1	0.15
aa	4	12.5±0.5	14.0±1.0	0.79	6	72.4±31.6	74.4±26	0.94	1.5±1.5	2.0±14.2	0.98
TaqI											
TT	8	62.2±31	57.8±30	0.047	7	13.7±3.0	19.3±1.2	0.14	-4.4±2.2	5.6±2.0	0.02*
Tt	15	48.2±15.1	23.2±8.4	0.072	13	37.3±13.7	57±15.7	0.17	-25±13.8	19.7±12.8	0.03*
tt	3	12.6±1.4	16.0±4.5	0.84	6	91.6±34.8	99.5±32	0.66	3.4±5.8	7.8±14.9	0.84

Values are Mean±SD;

* p value < 0.05;

a Absolute treatment effect is the absolute change from baseline to follow-up in the treatment group minus the absolute change from baseline to follow-up in the placebo group;

b P values for difference between treatment group and placebo group;

c P values for difference between follow-up visit and baseline visit; TAC, total antioxidant capacity; VDR, vitamin D receptor.

*Study design*

This study was an 8 week parallel, randomized, double-blind, placebo-controlled trial of vitamin D_3 _supplementation in breast cancer women. Participants were assigned to 2 treatment arms: placebo and vitamin D_3_ through a 2 months intervention period. Randomized block design method was exerted for grouping to receive either 50,000 IU/week (provided by Zahravi Pharm. Co., Tabriz, Iran) or edible paraffin as placebo. All capsules were indistinguishable in shape and color. Each subject was sequentially assigned a number upon study entry while all participants and investigators were blinded throughout the study. Recruitment began from March to April 2015 and the sampling was completed in September. At the beginning, gave their written informed consents, completed the questionnaires and were interviewed. Five milliliters of serums were collected from all subjects. Compliance was checked by calling the patients every week. Subjects were visited by the oncologist at the beginning and at 8th week post-intervention. After two months, blood samples were re-collected for measurement of serum 25 (OH)D concentrations and VDR polymorphism. Blood samples were drawn into two tubes for serum and CBC (complete blood count) and then immediately placed on ice and covered from light. Serum and plasma samples were separated by centrifuge with 2,000 RMP for 16 min using a 46H centrifuge (HETTIC, FRANCE). The serum samples were stored at -70 ^o^C freezer for further analyses. The levels of 2 different serum cytokines (TNF-α, TGF-β1) and total antioxidant capacity were measured at the baseline and the final visit by ELISA (enzyme-linked immunosorbent assay) method. VDR polymorphism including ApaI ،BsmI ،FokI, and TaqI of all subject was genotyped by SSP-PCR (Polymerase Chain Reaction with Sequence-Specific Primers) technique. The four SNPs of VDR are located in exon 2, intron 8, intron 8 and exon 9 of chromosome 12 (12q12-q14), respectively. Mean of 3-day energy and nutrients intake were analyzed using Nutritionist IV Database Manager 4.0 software. A trained nutritionist performed all data entrance.

*Laboratory Tests*

ELISA kits of bioactiva diagnostica GmbH Company (Homburg, Germany) were used to analyze serum TNF-α, TGF-β1 and TAC according to the manufacturer’s protocol. Different VDR gene polymorphisms were assessed by SSP-PCR technique (Lombard et al., 2006; Søborg et al., 2007). DNA was extracted by using commercial DNA extraction Kit (Roach, USA) from blood specimens. PCR reaction was implemented. After that, the product of PCR reaction electrophoresis was done on Agarose gel 1.5%. Allele frequencies and genotype polymorphism of the VDR gene were determined. Treatment groups were compared for basic characteristics and endpoint. Mean 25(OH)D concentrations were measured for the both groups at baseline and after 2-month follow-up.

Medical Research Ethics Committee at the Ahvaz Jundish apur University of Medical Science approved the study protocol. All participants signed the written informed consent.

*Statistical analysis*

Both treatment groups were assessed for comparing the characteristics at baseline and final follow-up visit using the Chi suare sample t for categorical variables and Independent t-test and paired T-test for continuous variables. The normality checked by using the Mann-Whitney test and Wilcoxon. Treatment effects were evaluated by assessing the differences in TNF-α, TGF-β1, and TAC concentrations from baseline to 2-month follow-up between the treatment and the placebo groups. All statistical analyses were performed with SPSS version 24. A P value of less than 0.05 was considered statistically significant.

## Results

A total of 125 patients were screened that led to the enrollment of 56 eligible patients; patients were randomly assigned to receive vitamin D_3_ (n=28) and placebo (n=28) for 2 months. The trial ended in September 2015. Four patients (8%) withdrew from the study: 2 patients in the vitamin D_3_ group and 2 in the control group due to side effects (n = 1), unwillingness to continue participation (n = 1), diagnosing metastasis (n =1), and consuming Ca/vitamin D pills (n = 1). Consequently, 26 patients in the vitamin D_3_ group and 26 patients in the placebo group finished the trial. Compliance rate was 95.6% patient in the vitamin D_3_ group and 91.6% patients in the placebo group and it was not significant. Baseline characteristics of the participant were similar between the supplemented and placebo groups (Table 1).The mean age of the participants was 47±8.8 y. From all subjects 49% were Arab and 51% were Fars. The dietary intake of participants at baseline and 2 months after supplementation using three 24-hour recalls indicated no significant differences between the groups. 

As indicated in Table 2, the mean of 25(OH)D increased from 28 to 39 ng/mL in vitamin D group (P = 0. 004). The mean of TAC decreased from 45.2 to 29.2 U/ml in the placebo while it increased from 48.9 to 63.5 U/ml in vitamin D group. These increase and decrease were significant. Accordingly, the mean of increase was 14.6 U/ml in supplemented group and mean of reduction was 16 U/ml in placebo group (p≤0.04). The effect of vitamin D_3 _on the both inflammatory markers i.e. TNF-α, TGF-β1 was not significantly different compared to the placebo group. 

Individuals were classified according to their VDR genotypes to evaluate the changes in the inflammatory factors TNF-α (Table 3) and TGF-β1 (Table 4) and TAC (Table 5). The effects of vitamin D_3_ on TNF-α, TGF-β1 of the most reported VDR subgroups showed degrees of decrease, however, changes were not statistically significant (Table 3, Table 4). Supplementation significantly increased the serum TAC levels in TT, Tt cases (p ≤0.03) but not tt subgroup. Additionally, the serum TAC levels significantly elevated in Ff individuals (p = 0.036) but not FF/ff subjects. Despite medium increment of TAC among AA genotype, no significant changes were found in the subgroup (P=0.06). The other variables did not significantly change.

## Discussion

This study examine the impact of oral high-dose vitamin D_3_ supplementation on circulating inflammatory markers and TAC according to four VDR polymorphisms (i.e. BsmI, ApaI, TaqI, and FokI) in women with breast cancer. Serum levels of 25(OH)D and TAC elevated in treatment group. Despite a reduction trend seen in TNF-α, and TGF-β1 concentration, we did not find any significant impacts of vitamin on these biomarkers. Also, the serum TNF-α, and TGF-β1 levels did not show any differences with regard to VDR genotype following supplementation. However, TAC concentrations of individuals with the TT/Tt, and Ff genotypes significantly elevated post-intervention.

The interaction of supplementation and inflammatory markers was evaluated. Available evidence is extremely limited about regulating role of vitamin D on serum cytokines among breast cancer women. Our results are similar to another study by Jorde et al., (2010) that found no changes in inflammatory markers following vitamin D supplementation of 20,000 IU/week or 40,000 IU/week of D_3_ in overweight subjects. Chandler et al., (2014) also observed in African Americans that using three doses of vitamin D_3_ including 1,000, 2,000, or 4,000 IU/day orally for 3 months could not reduce the CRP, IL-6, IL-10, and sTNF-R2 (soluble TNF receptor 2) levels after supplementation period. On the contrary, one-year supplementation with vitamin D decreased serum IL-6 levels while hs-CRP (high sensitivity- CRP) levels were significantly increased and TNF-α did not respond to supplementation in overweight subjects (Beilfuss et al., 2012). Administration of vitamin D_3_ doses of 2,000 IU per day for three months did not affect 10 different cytokines (IL-2, 4, 5, 6, 8, 10, 13, IFN-γ, TNF-α) in healthy adults (Yusupov et al., 2010). A significant reduction was observed in the serum TGF-β levels at the 8th week in vitamin D deficient PCOS women who received 50,000 of vitamin D_3_ supplementation once weekly (Irani et al., 2015).

Researches on cancer patients have reported different findings. Serum inflammatory markers may moderately be a nonspecific measurement of short-term differences in tissue-specific inflammatory pathways that are importance in breast cancer (Reeves et al., 2011). Some RCTs have indicated significant favorable effects of vitamin D supplementation on inflammatory cytokines such as IL-6, sTNF-R2, and CRP, but only in precisely chosen groups of diabetics patient (Shab-Bidar et al., 2012) and patients with colorectal adenoma (Hopkins et al., 2011). Additionally, The role of TGFβ-1 in cancer progression has been shown to be multifaceted, depending on the tumor stage (Parvani et al., 2011). This cytokine acts as a potential growth inhibitor during beginning and development of cancer. TGF-β1 is also regarded as a metastasis stimulator, contributing to malignant progression (Zarzynska, 2014). Surprisingly, the majority of carcinogenic cells lose sensitivity to the inhibitory effect and initiate to secrete TGF-β1 when entering the phase of uncontrollable growth (Boulanger et al., 2005). This may be the reason for observing no significant changes in blood concentration of TGF-β1.

Vitamin D deficiency or insufficiency is prevalent among women with breast cancer indicating that dietary intake and sun exposure are not sufficient for maintaining adequate levels of this vitamin (Alipour et al., 2014; Bidgoli and Azarshab, 2014). This deficiency might be related to TAC levels and its function (Gargari et al., 2016). The Endocrine Society (2011) has suggested that supplementation doses should be 50,000 IU of vitamin D_2_ or D_3_ for vitamin D deficient individuals (Holick et al., 2011). A recent meta-analysis revealed that vitamin D_3_ is more capable of improving serum 25(OH)D_3_ concentrations when administering as a high oral dose (50,000 IU single dose or 300,000 IU single dose, and 50,000 IU/month) in comparison to prescription of daily dose of D_2_, but the influence was lost with 1,000-4,000 IU/d supplementation (Tripkovic et al., 2012). A retrospective study showed weekly high-dose supplementation statistically improved 25(OH)D_3 _levels Compared to the no-supplementation group though daily low-dose supplementation was failed to increase 25(OH)D_3_ levels significantly. This study involved women with stage 1–3 breast cancer who have different cancer treatment (radiotherapy, chemotherapy, and/or hormone therapy).

In the line with our study, de Medeiros et al., (2015) showed that 4 weeks of supplementation with 200,000 IU of vitamin D_3_ administered as a single dose significantly improved serum 25(OH)D levels and total antioxidant capacity in elderly women with vitamin D insufficiency. Similar results have been indicated in diabetic patients who took vitamin D_3_-fortified butter-milk containing 170 mg calcium and 500 IU/250 ml twice a day for 12 weeks (Shab-Bidar et al., 2015b). In contrast to ours, some studies did not find any effects of vitamin D supplementation on TAC. In diabetic women, supplementation with 50,000 IU vitamin D_3_ for 6 weeks did not affect TAC concentrations (Asemi et al., 2013). Similar result was observed in pregnant women who consumed 50,000 IU vitamin D for 6 weeks from weeks 24–28 of pregnancy up to delivery (Asemi et al., 2014). Some studies prescribed combination of vitamin D_3_ with other dietary supplements including vitamin C and calcium. This co-administration improved Glutathione (GSH) activity (Ekici et al., 2009) and decreased oxidative DNA damage in the normal colorectal mucosa (Fedirko et al., 2010). It is proposed, simultaneous prescription of them may lead to the supplementary benefit in TAC increment. 

The results are controversial about the impact of vitamin D supplementation on inflammatory markers and TAC with regard to VDR genes. These incompatible outcomes may be attributable to the effect of genetic polymorphisms regulating the response to supplementation (Gagnon et al., 2014). VDR gene variation may be the reason of different response to vitamin D (Barry et al., 2014). We showed that individuals with the VDR FokI Ff, TaqI TT/ Tt genotypes had significantly higher TAC in responses to vitamin D_3_ supplementation. Unexpectedly, serum levels of inflammatory markers in subjects with different genotype had no significant changes. On the contrary, de Medeiros showed among elderly women with BB/Bb genotype had lower levels of us-CRP post intervention parallel with increment of TAC levels (de Medeiros Cavalcante et al., 2015). We hypothesized that the elevation of TAC concentrations with vitamin D_3_ supplementation might be effective in inhibition of oxidative stress in the subgroup of patients with FokI Ff, TaqI TT/ Tt while not effective in patients with the FokI ff, TagI tt, ApaI, BsmI genotypes in our population.

Vitamin D has long been known for its important role in calcium and phosphate homeostasis. Recently, It was demonstrated that receptors of vitamin D decrease the activity of NF-κB (nuclear factor kappa-light-chain-enhancer of activated B cells), the pro-inflammatory transcription factor. This note demonstrates that vitamin D nuclear receptor plays a crucial role in the innate immune response (Adorini et al., 2007; Szeto et al., 2007). Several researchers have shown that the VDR genotype may control the inflammatory marker profile and the anti-inflammatory response to treatment with vitamin D. A clinical trial has demonstrated that the VDR BsmI BB genotype may change the inflammatory marker profile and the anti-inflammatory response to treatment with vitamin D (Shab-Bidar et al., 2015b). Cachectic cancer patients with TT and bb genotype were higher CRP levels than tt/Tt and BB/Bb genotype (Punzi et al., 2012). In contrast, the oxidative stress parameters including MDA (malondialdehyde), GSH and TAC in type 2 diabetic subjects treated with vitamin D for 12 weeks showed no significant differences in FokI genotype, although ff variant subgroup showed the weakest response to vitamin D (Shab-Bidar et al., 2015a).

Some drawbacks of our study need to be mentioned. Due to some limitations, we could not assess the effect of vitamin D supplementation on other biomarkers of inflammation, including hs-CRP, IL-1, and IL-6, as well as enzyme superoxide dismutase, glutathione, and malondialdehyde. The sample size is relatively small and further research with larger sample size should be done.

Our findings indicated that individuals with VDR TT/Tt, and Ff genotype can be regarded as the high responders to vitamin D supplementation regarding body total antioxidant capacity. The prevalence of different VDR variants among breast cancer women could explicate, in part, certain inconsistencies seen in the response to vitamin D in several markers of breast cancer. Further studies are needed to follow this intervention in larger populations, on different biomarkers, and other racial groups to illuminate chemopreventive potency of vitamin D on inflammation, oxidative stress markers and genetic interactions.
